# Peritoneal Dialysis-Induced Encapsulating Peritonitis: Diagnostic and Therapeutic Challenges in Women with Benign Gynecological Pathology

**DOI:** 10.3390/jcm13102921

**Published:** 2024-05-15

**Authors:** Cristian Iorga, Cristina Raluca Iorga, Iuliana Andreiana, Simona Hildegard Stancu, Traian Constantin, Victor Strambu

**Affiliations:** 1 Faculty of Medicine, “Carol Davila” University of Medicine and Pharmacy, 050474 Bucharest, Romania; cris.iorga@yahoo.com (C.I.); iuliandreiana@yahoo.com (I.A.); simonastancu2003@yahoo.com (S.H.S.); drstrambu@gmail.com (V.S.); 2 Surgery Clinic, “Dr. Carol Davila” Clinical Nephrology Hospital, 010731 Bucharest, Romania; 3 Nephrology Clinic, “Dr. Carol Davila” Clinical Nephrology Hospital, 010731 Bucharest, Romania; 4 Department of Urology, “Prof. Dr. Th. Burghele” Hospital, 050652 Bucharest, Romania

**Keywords:** peritoneal sclerosis, encapsulating peritonitis, emergency surgery

## Abstract

**Background:** Peritoneal sclerosis (PS) and its most severe form, encapsulating PS (EPS), are rare entities that can occur in various procedures (liver transplantation, intraperitoneal chemotherapy) or secondary to medications (beta-blockers); however, PS or EPS typically occur in patients undergoing peritoneal dialysis as a form of renal function substitution. Medical or surgical treatments can be applied, but morbidity and mortality have high rates. This condition typically presents clinically as an intestinal obstruction caused by the inclusion of the intestinal loops in the peritoneal fibrous membrane. **Methods**: Herein, we present data from a single tertiary surgery center that has dedicated teams for patients receiving dialysis. Over 12 years, we analyzed a group of 63 patients admitted for catheter replacement/removal or for acute surgical pathology. In five cases (7.9%), we diagnosed EPS. Two patients with EPS presented with atypical abdominal pathologies requiring emergency surgery: one case of hemoperitoneum caused by a ruptured ovarian cyst and one case of uterine fibroids and metrorrhagia. **Results**: The definitive diagnoses were established intraoperatively and by analyzing the morpho-pathological changes in the peritoneum. The possible intraoperative challenges included laborious dissection, difficulties in restoring the correct anatomical landmarks, an increased duration of the surgical intervention and a high rate of incidents and accidents. **Conclusions**: The aim of the present study was to emphasize the possibility of other surgical pathologies overlapping with EPS, increasing the complexity of the surgical intervention.

## 1. Introduction

Encapsulating peritoneal sclerosis (EPS) is a rare entity typically occurring in patients receiving peritoneal dialysis (PD) and carries significant morbidity and mortality rates [1,2,3]. The first description of EPS dates from 1907 when Dr. Owtschinnikow described as peritonitis chronica fibrosa incapsulata [4]. Subsequently, in 1978, EPS was referred to as the abdominal “cocoon”, and in 1980, EPS was found to be associated with PD [5,6]. However, EPS has also been described in other conditions such as abdominal or gynecological cancer, endometriosis, cirrhosis, organ transplantation, drug administration, infection, systemic rheumatologic and inflammatory diseases, mechanical and chemical intraabdominal irritants or as primary EPS with unclear etiology [7].

For patients administered PD, the etiology of EPS is multifactorial. The major and unanimously recognized risk factor is duration of PD [8,9,10,11,12,13,14]. There is a direct correlation between PD duration and EPS occurrence.

The disease is rarely encountered in the first 3 years of dialysis, and after 5 years, the incidence of EPS remains low but increases continuously. Notably, in two-thirds of patients, symptoms of EPS appear months or even years after the transfer to hemodialysis or kidney transplant. This feature along with different transplant or hemodialysis access policies explains the variable incidence of EPS reported by registries [1,3,9].

Several studies suggest that another important risk factor is represented by peritonitis episodes (bacterial, particularly with Staphylococcus aureus and Pseudomonas aeruginosa, or fungal) [15,16,17,18,19]. However, in some studies there is no correlation between peritonitis and EPS. Korte et al. did not find a direct link between EPS and peritonitis episodes [20]. Similarly, Johnson did not correlate the frequency or number of peritonitis episodes with EPS [3]. In a US study, Gayomali demonstrates that peritonitis is not a condition per se of EPS; rather, the microorganisms involved and the duration of peritonitis episodes correlate with EPS [21].

PD alone leads to chronic inflammation and changes in the peritoneal membrane (first hit). Morphological and functional changes in the peritoneal membrane are induced by prolonged contact with dialysis solutions [22].

The dialysis catheter itself can be a trigger for inflammation, either directly—a foreign body reaction—or by creating a bacterial biofilm [23,24].

Peritonitis episodes act as a second hit, along with kidney transplantation, hemodialysis (HD) transfer and acute intra-abdominal pathology, which overlap on the pre-existing proinflammatory background.

Renal transplantation and HD transfer imply the acute cessation of PD. In addition, the case of renal transplantation associated with the action of immunosuppressive therapy (tacrolimus, cyclosporine) is recognized to induce peritoneal fibrosis and angiogenesis [25,26].

Even if the acute cessation of PD is considered itself to be a risk factor, there are studies not supporting this theory [13].

Other risk factors have also been studied, such as the use of certain drugs, disinfectant solutions containing chlorhexidine or povidone-iodine and genetic predisposition, but the results are inconstant [27,28,29].

An alteration in the peritoneal membrane induces ultrafiltration failure (UFF) which can be used as a prognostic criterion for EPS. Alatab et al. showed that maintaining patients on PD treatment despite UFF increases the risk of EPS occurrence [19].

Clinical manifestations of EPS consist of signs and symptoms of intestinal dysfunction (such as abdominal pain, bloating, nausea, vomiting, weight loss, malnutrition and total bowel obstruction in the late stages) sometimes associated with ultrafiltration failure, bloody effluent or recurrent sterile peritonitis. These symptoms are chronic, insidious and non-specific but are essential for diagnosis, as they raise the suspicion in predisposed patients [16].

A positive diagnosis of EPS is established based on the aforementioned clinical signs in conjunction with CT scan images that show loculated ascites, calcifications and the thickening of the parietal and visceral peritoneum [30]. The direct visualization of the abdomen and histology can also be used to establish a diagnosis but are rarely needed. Several types of lesions have been described: the peritoneal surface is reduced to a thickened and rough membrane, compared by some authors to the sole of a worn shoe [10], and the thickening of the peritoneum can be cellular (through the activation of fibroblasts) or acellular (likely through interstitial collagen deposits) [11]. The thickening of the peritoneal membrane determines the rigidity and thickening of the intestinal serosa and subsequently the decrease in intestinal motility until it has disappeared, culminating with intestinal occlusion. The mesentery, stomach, liver and spleen are also affected by sclerosis, which also characteristically does not affect the entire abdomen to the same degree, and only the most affected areas cause the appearance of the abdominal “cocoon” [10,12].

Tamoxifen and/or steroids administered in the early stages and extensive surgical adhesiolysis in the late stages significantly ameliorate the morbidity and mortality of EPS [31].

The purpose of this study is as follows. Although intestinal obstruction is the common manifestation that results in the referral of patients with EPS to a surgeon, there have been cases that required emergency surgery for reasons other than EPS. We outline out the diagnostic and therapeutic challenges in two cases of women with benign gynecological pathology overlapping with EPS.

## 2. Materials and Methods

A group of 66 patients who underwent PD and were admitted to the General Surgery Department of “Dr. Carol Davila” Teaching Hospital of Nephrology (Bucharest, Romania) for catheter replacement, catheter removal or surgical co-morbidities from November 2011 to March 2021 were analyzed.

Our hospital is a tertiary center specializing in adult nephrological patients, who require nephrological treatment or surgical interventions. Our team consists of surgeons, nephrologists, anesthesiologists, pathologists and radiologists specialized in the care of chronic kidney disease patients.

Inclusion criteria for the patients in the study group were as follows:

− Patients on PD requiring surgical intervention (as above);
− Patients with complete personal data extracted from medical records;
− Patients that could be followed up at least 6 months postoperatively;

Exclusion criteria were as follows:

− Incomplete essential personal data (particularly referring to missing anatomopathological result, duration of PD and type of PD solution);
− Less than 6 months follow-up.

From the 66 patients extracted from the electronic records, we included 63 patients in the study (respecting inclusion and exclusion criteria). One patient was excluded due to lack of follow-up for at least 6 months postoperatively, and two patients were excluded due to incomplete data (duration of PD, missing anatomopathological result).

All patients in the study group were on PD using glucose-based solutions.

All patients requiring surgical intervention were switched to hemodialysis, temporarily or definitively. 

In cases of EPS diagnosis, the PD was discontinued definitively.

The demographic and clinical parameters of the included patients were retrospectively extracted from the medical electronic records. In all EPS cases, samples were collected from the peritoneum for pathological examination. All samples were analyzed by the same pathologist.

Statistical analyses were performed using the Analyse-it™ Standard Edition (Analyse-it 4.80 Software, Ltd., Leeds, UK) package. Categorical variables are presented as percentages, comparisons of which were performed using Pearson’s χ^2^ test. A *p* ≤ 0.05 was considered statistically significant.

Continuous variables are presented as median and quartiles [1; 3], comparisons of which were conducted using the Kruskal–Wallis test.

## 3. Results

All 63 patients (44% women; median age, 62 [48.0; 70.8] years) were treated with continuous ambulatory PD. Catheter removal for scheduled transfer to hemodialysis and for refractory PD-associated infections were the two highest causes for surgical intervention (52.4 and 27.0%, respectively). An additional 11% of interventions were for catheter reposition, 4.7% for co-morbidities, 3.2% for EPS and 1.6% for catheter removal following a successful kidney transplant. The same team performed all surgeries. Of the 63 patients, 5 (7.9%) had a final diagnosis of EPS. Table 1 shows the clinical characteristics and morpho-pathological changes of these 5 patients with EPS. 

Following univariable analysis, it was demonstrated that patients with EPS were younger (median age, 39 [30; 52] vs. 63 [50.8; 71.2] years; *p* = 0.0019) and had received PD for longer (114 [86; 132] vs. 18 [11; 46.5] months; *p* = 0.0013) compared with patients without EPS. The number of patients having at least one previous episode of peritonitis was similar between the two groups, but the percentage difference is double (80% in the EPS group and 39.7% in non-EPS group, *p* = 0.08). In this case, the influence of previous peritonitis has no statistical significance as a risk factor for EPS. Of the 5 patients with EPS, 2 required emergency surgery for gynecological conditions, the further details of which are described below.

**Figure 1:**
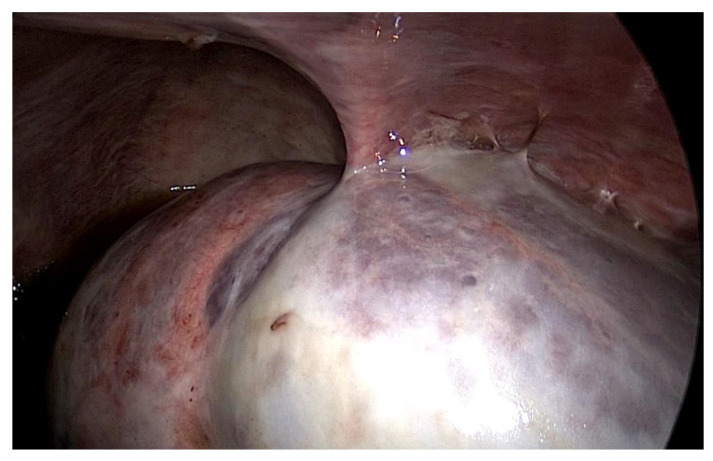
Intraoperative finding: thickened peritoneum.

The first patient was a 25 year old woman with lupus nephritis who had undergone PD for 10 years. The PD history of the patient was associated with three episodes of peritonitis (including *Mycobacterium tuberculosis*, *Enterococcus faecalis* and *Staphylococcus aureus* infections, the last of which was 6 years before presentation) and negative ultrafiltration for several months requiring four hypertonic exchanges daily (2X 1.36% and 2X 3.86% Dianeal PD Solution (Baxter International)) to control overhydration. For several months, the patient was suffering from metrorrhagia and bloody effluent for several days. During hospitalization, the effluent became notably hemorrhagic with acute anemia. Emergency surgery was performed revealing hemoperitoneum due to an adnexal pathology. Intraoperatively, we found extensive peritoneal fibrosis encapsulating the small bowel loops and the organs located in the pelvis in a “cocoon” (Figure 1). Furthermore, there was significant bleeding from the site where the right adnexa should have been located and the right fallopian tube and right ovary could not be identified. A visceral “block” representing the right adnexa was dissected and isolated with great difficulty, which was resected to stop the source of the intraperitoneal hemorrhage. A final diagnosis of EPS was confirmed by the pathologist. The immediate and late postoperative outcomes were good, without any surgical complications; however, the patient subsequently died 6 months later due to a hemorrhagic stroke.

**Figure 2:**
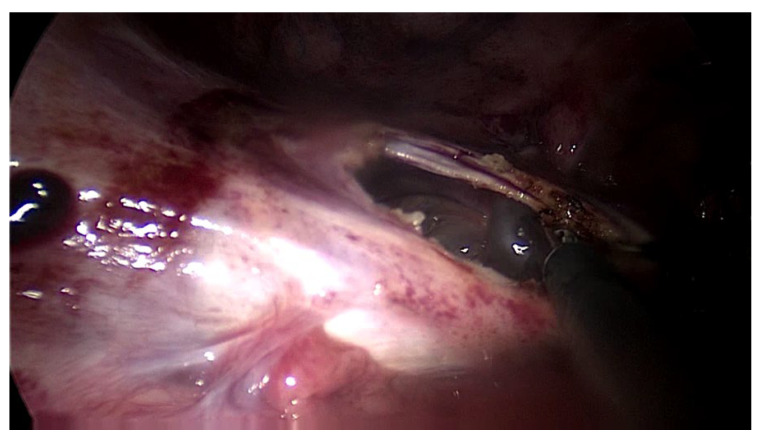
Intraoperative finding: thickened peritoneum, ascites.

The second patient was a 44 year old woman who had undergone PD for 11 years. The patient was referred for nausea, vomiting and metrorrhagia accompanied by a palpable mass in the lower abdomen. A CT scan revealed the presence of an abdominopelvic tumor measuring 12 cm in diameter, suggestive of a uterine fibroid causing the compression of the rectum. A hemostatic curettage was performed in an attempt to postpone surgery, which proved unsuccessful. Then, laparotomy and a definitive transfer to hemodialysis treatment was decided. The intraoperative macroscopic appearance of the parietal and visceral peritoneum revealed only some areas of peritoneal fibrosis, except for the pelvic peritoneum, which showed extensive fibrosis (Figure 2). The fibromatous uterus was encapsulated in a pelvic “cocoon”, and the rectosigmoid junction adhered to this “pelvic block”.

The procedure then consisted of a total hysterectomy with bilateral adnexectomy. The adhesiolysis of the rectosigmoid junction from the pelvic block was performed with great difficulty, with the surgical procedure lasting 4.5 h. The patient had a slow postoperative recovery, as it was marked by a difficult resumption of the gastrointestinal transit.

**Figure 3:**
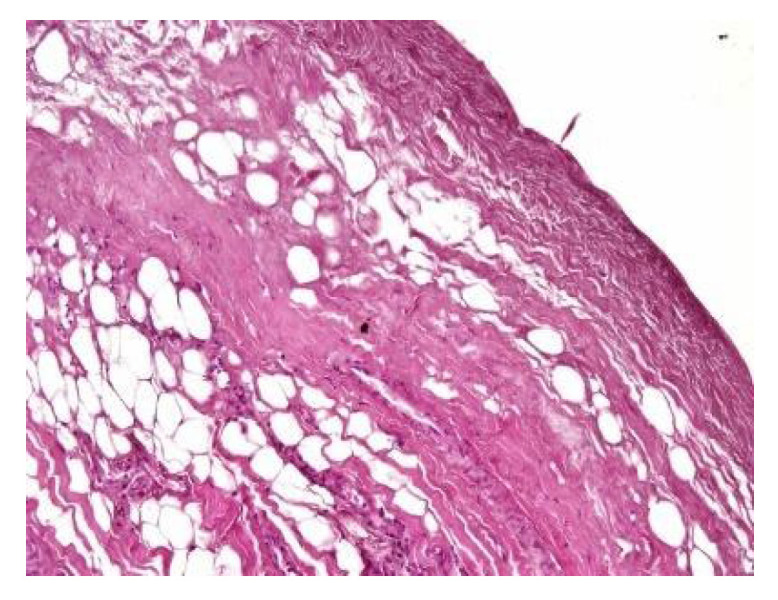
Lamellar fibrosis, condensed fibrin on the surface of the peritoneum and decreased cellularity (hematoxylin and eosin staining, original magnification × 100).

**Figure 4:**
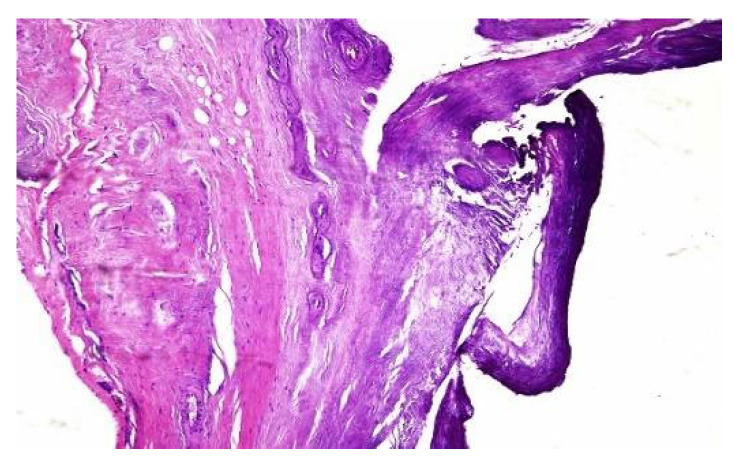
Lamellar fibrosis, condensed fibrin on the surface of the peritoneum and decreased cellularity (hematoxylin and eosin staining, original magnification × 100).

**Figure 5:**
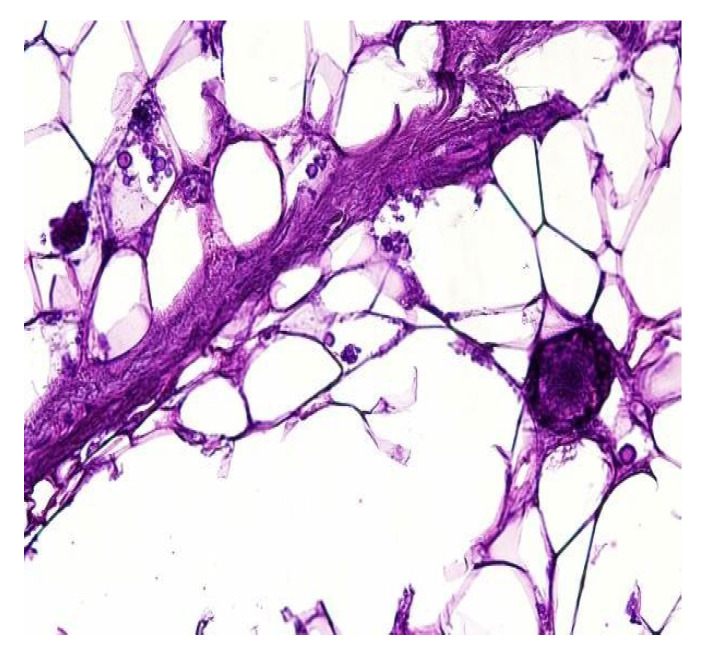
Dystrophic calcifications in the peritoneum (hematoxylin and eosin staining, original magnification × 100).

**Figure 6:**
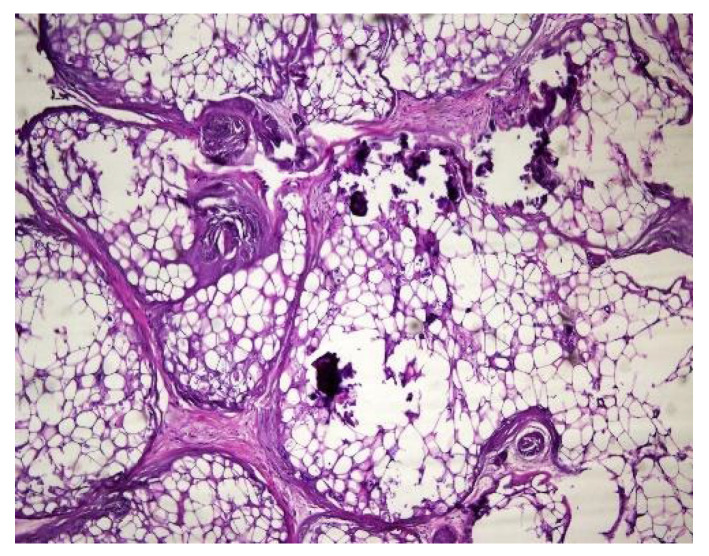
Dystrophic calcifications in the peritoneum (hematoxylin and eosin staining, original magnification × 100).

In both of the described cases, optical microscopy revealed lamellar fibrosis, deposits of fibrin on the surface of the peritoneum (Figure 3 and Figure 4) and dystrophic calcifications in the peritoneum (Figure 5 and Figure 6).

All 5 patients with EPS underwent extensive adhesiolysis, 2 of which (patients 1 and 5) also received prednisone (0.5 mg/Kc daily for 1 month, which was then progressively decreased). The immediate postoperative period (7 days after surgery) was without complications and the patients were discharged.

## 4. Discussion

Herein, we present data from a single tertiary surgery center that has dedicated teams for patients receiving dialysis. Extensive adhesiolysis for EPS was performed in almost 8% of the surgical patients receiving PD. In agreement with the literature, these patients were young and had a long duration of treatment [20]. All patients had suggestive symptoms for several months, which were not recognized by their physicians. In Romania, the prevalence of PD is very low (1.7%, 10–15 patients/dialysis unit). Therefore, a physicians’ lack of experience is one of the major factors that negatively influences the prognosis of patients receiving PD and most likely explains the lack of recognition of early EPS symptoms in more than half of the presented patients in this study (overhydration in patients 1 and 4, nausea and vomiting in patient 2) [32].

The aim of the present study was to emphasize the possibility of other surgical pathologies overlapping with EPS, increasing the complexity of the surgical intervention. To the best of our knowledge, the association between EPS and benign gynecological pathology in patients receiving PD has not yet been described. When searching the literature, we found only one case report of a 66 year old non-nephrological woman who was hospitalized for diarrhea and abdominal distension. Extensive work-up revealed ascites, bulky ovaries, a fibroid uterus and multiple peritoneal and mesenteric deposits. During laparotomy, the bowel was firm and solid with no obvious peristalsis due to serosal fibrosis, and histology showed a fibrous process with spindle cells and inflammation. After a hysterectomy, the patient remained symptom-free, and a normal CT image was observed after 6 months of follow-up [33]. The morpho-pathological changes were similar to those observed in the two female patients with gynecological pathology (“iced” abdomen due to extensive fibrosis and histology with low-grade inflammation) described in the present study; however, these two patients had more severe disease with bone metaplasia and a pelvic “block” encapsulating all internal genitalia. Moreover, this last feature was the single morpho-pathological feature that differentiated these patients from the other 3 patients receiving PD, raising the question of a contribution of pelvic pathology with EPS severity in association with long PD treatment.

Several studies have attempted to identify and/or standardize the morpho-pathological changes in EPS, differentiating it from PS, which is a common occurrence in patients receiving PD and presents as a simple sclerosis [12,13,22,34,35]. The most frequent aspects observed were fibrin deposits, fibroblast swelling and mononuclear cell infiltration [11,12,22]. Garosi et al. investigated 39 biopsies from patients with EPS and found that tissue and arterial calcification, the thickening of the submesothelial layer and vasculopathy were the most significant observed changes [30]. In another study, Sherif et al. found that only fibrin deposits and the thickening of the compacta were significant [36]. In the present study, lamellar fibrosis, decreased cellularity, low grade perivascular inflammation and dystrophic peritoneal calcifications were frequent histological changes. Similarly, Braun et al. attempted to standardize the lesions and to define reproducible histological parameters in patients with EPS. It was found that calcification was a highly indicative criteria for EPS. Furthermore, mesothelial denudation, chronic inflammation, fibrin deposits, decreased cellularity and the presence of fibroblast-like cells were also indicative of EPS [10].

According to the “two hit” hypothesis of EPS pathogeny, peritonitis episodes, transfer to hemodialysis, kidney transplant and acute intra-abdominal pathology are triggers (second hit) for peritoneal inflammation that is already in an inflammatory state (first hit) due to chronic exposure to PD solution [37]. Supporting this theory, Wong et al. found that 1 in every 5 patients receiving long-term PD with catheter removal for refractory peritonitis (19.5%; median duration of therapy, 71.6 ± 43.3 months) evolved to EPS in a 6 month period of observation. This was explained by the presence of two important “second hit” factors (bacterial peritonitis and the withdrawal of dialysis) [38]. In the present study, all patients had possible “second hit”-type events before EPS symptoms presented (peritonitis, hemodialysis transfer, hemorrhagic ovary cyst and uterine fibroid), but due to the small number of patients, an analysis of possible associations was not possible.

The surgical treatment of EPS has extensively changed over the years and improved survival [1,39,40]. Treatment mainly consists of adhesiolysis and the removal of the encapsulated membrane, hence clearing the bowel loops. However, the treatment is still not standardized and data from the literature show a post-surgery survival rate of 61–100% [14,39,40,41]. The main cause of death is uncontrolled peritonitis during postoperative care. In addition, bowel resection is associated with an increase in postoperative morbidity and mortality; therefore, in specialized centers, this procedure is avoided as much as possible [3,12,13]. Almost one-quarter of patients have recurrent disease in the first 2 years following surgery [41]. Unfortunately, long term follow-up data was only available for 2 patients in the present study. These patients received steroids with no relapse during the 6 months of follow-up. We consider that medical treatment after surgery could further ameliorate prognosis by lowering the chances of relapse, but this requires further research.

## 5. Conclusions

In conclusion, in the present study, we aimed to describe a very rare condition, EPS, that affects patients undergoing PD and that leads to intestinal obstruction. We presented two cases of patients whose clinical manifestations of EPS were underrecognized. Intraoperative exploration for other acute pathologies and histological findings established the definitive diagnosis.

## Figures and Tables

**Table 1:** Clinical and morpho-pathological characteristics of the EPS patients.

	Age (Years)	Gender	PD Duration (Months)	Peritonitis No.	Referral Diagnosis	Clinical Manifestation	Morpho-Pathologic Changes
P1	25	F	124	3	metrorrhagia for 6 months bloody effluent for days	metrorrhagia nausea, vomiting for months	- pelvic “cocoon” - bleeding right adnexa - unidentified right adnexa in the pelvic “block”
							- lamellar fibrosis - low cellularity - deposits of fibrin on the surface of the peritoneum - dystrophic peritoneal calcifications
P2	44	F	132	0	metrorrhagia for 8 months	metrorrhagia overhydration for months	- focal abdominal fibrosis- pelvic “cocoon” encapsulating the uterus and recto-sigmoid junction - ascites
							- lamellar fibrosis - deposits of fibrin on the surface of the peritoneum - dystrophic peritoneal calcifications
P3	39	M	114	yes	bowel obstruction	abdominal pain, bloating for four months, immediate after transfer in HD bowel obstruction for 5 days	- thick peritoneum - entero-enteral and entero-parietal adherence
							- fibrosis - hyaline collagen transformation
P4	31	F	64	1	transfer in HD	overhydration for 4 months after peritonitis	- entero-enteral and entero-parietal adherence - abdominal “cocoon” - 3 loculated cysts
							- fibrosis - peri-vascular infiltration with inflammatory cells - bone metaplasia
P5	68	M	132	6	bowel obstruction	anorexia nausea, vomiting severe anemia starting 10 months after transfer in HD	- entero-enteral and entero-parietal adherence - abdominal “cocoon”
							- fibrosis - dystrophic peritoneal calcification

EPS—encapsulating peritoneal sclerosis; HD—hemodialysis; No.—number; P—patient; PD—peritoneal dialysis.

## Data Availability

Data are contained within the article.
